# Structural basis for neutralization of an anicteric hepatitis associated echovirus by a potent neutralizing antibody

**DOI:** 10.1038/s41421-021-00264-3

**Published:** 2021-05-25

**Authors:** Rui Feng, Lei Wang, Dawei Shi, Binyang Zheng, Li Zhang, Hai Hou, Deju Xia, Lunbiao Cui, Xiangxi Wang, Sihong Xu, Kang Wang, Ling Zhu

**Affiliations:** 1 CAS Key Laboratory of Infection and Immunity, CAS Center for Excellence in Biomacromolecules, Institute of Biophysics, Chinese Academy of Sciences, Beijing, 100101 China; 2 University of Chinese Academy of Sciences, Beijing, 100049 China; 3 Institute for In Vitro Diagnostics Control, National Institutes for Food and Drug Control, Beijing, 100050 China; 4 National Health Commission of the People’s Republic of China, Key laboratory of Enteric Pathogenic Microbiology (Jiangsu Provincial Center for Disease Control and Prevention), Nanjing, 210009 Jiangsu China; 5 Institute of Medical Research, Northwestern Polytechnical University, Xi’an, 710072 Shaanxi China

**Keywords:** Cryoelectron microscopy, Molecular biology

Dear Editor,

Human enteroviruses (HEVs) are ubiquitous pathogens responsible for multiple human diseases, ranging from hand-foot-and-mouth disease (HFMD), meningitis, to poliomyelitis, encephalitis, etc^1^. HEVs comprise four main groups, Enterovirus A–D (HEV-A, B, C, and D), among which HEV-B is the largest group, including coxsackievirus A9 (CVA9), coxsackievirus B1–6 (CVB1–6), and over 30 serotypes of echoviruses. Echovirus 3 (E3), a serotype of HEV-B, first isolated in 1953, leads to highly contagious and severe diseases in humans, such as aseptic meningitis, myocarditis, and anicteric hepatitis^2^. Infection in neonates and infants within the first few weeks of life can be fatal. Currently, there are no approved vaccines or antiviral drugs for treating infections caused by viruses belonging to the HEV-B group.

Although the atomic structures for numerous HEVs have been studied^3–8^, large gaps in our knowledge concerning structural determinants for specificity between the serotypes/subgroups and immunogenic characteristics for serotype-specific or cross-reactive epitopes within HEV-Bs still exist. Therefore, an in-depth understanding of the structural, immunogenic features and key epitopes of E3 should be useful in providing guidance for the rational drug design against HEV-B infections. We obtained the E3 virus (genotype HNWY-01 strain) from the Jiangsu Provincial Center for Disease Control and Prevention (CDC) and propagated it in RD cells. Following rounds of purifications and analyses, three types of particles were identified, one containing significant amounts of viral RNA characterized as mature virus (or full particle, F-particle) and the other two being empty inside with substantially different sizes defined as expanded and compact empty particles (EE- and EC-particles) (Supplementary Fig. S1). Cryo-electron microscopic (cryo-EM) structures of the three types of particles: F-particle (~ 64%), EC-particle (~ 3%), and EE-particle (~ 33%) were reconstructed to 3.2 Å, 3.8 Å, and 3.1 Å resolution, respectively (Fig. 1a; Supplementary Figs. S2a and S3a–c and Table S1). The external surfaces of the F- and EC-particles with a diameter of ~ 325 Å are indistinguishable, apart from some disorder on the inside of the EC-particle, including N-termini of VP1 and VP0 (Fig. 1a). By contrast, the EE-particle exhibits a typical expanded form with a ~ 4.5% increase in diameter (~ 340 Å) and notable perforations at the icosahedral two-fold axes (Fig. 1a; Supplementary Figs. S4 and S5d). Superimposition of the asymmetric units (protomers) verifies the similarity in capsid structures of the F- and EC-particles and reveals substantially conformational differences between F- and EE-particles in several exposed loops, such as VP1 BC, DE, EF and HI loops, VP3 EF and GH loops (Supplementary Figs. S4 and S5a–c). Remarkably, most of these loops are key determinants for immunogenicity in HEVs, indicating that EC-particle, rather than EE-particle, has more potential as candidates for the development of a vaccine. Incorporation of EE-particles in the vaccine would require a rational strategy to facilitate the structural transition from the expanded to the compact state.

**Fig. 1:**
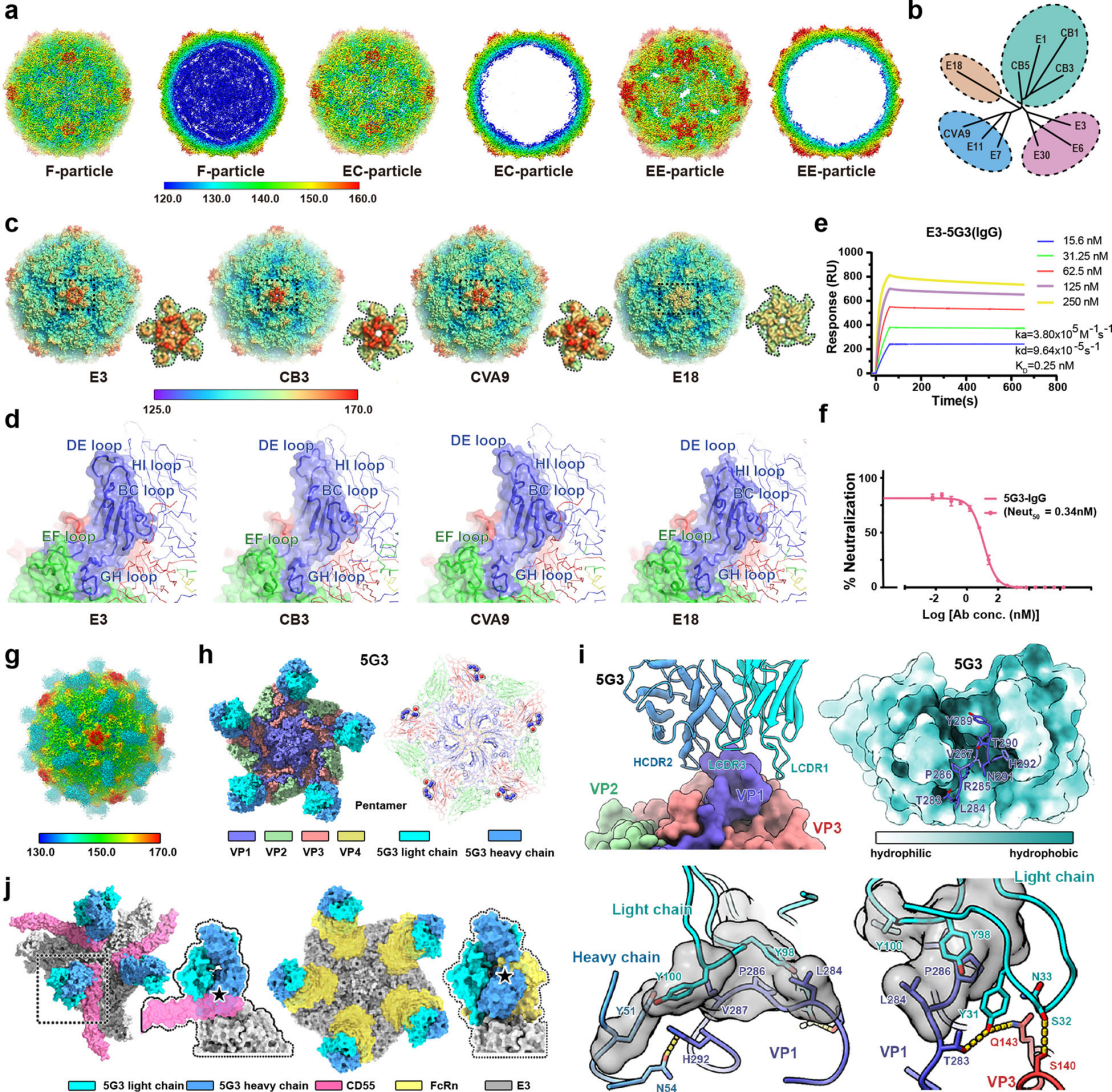
Structural, biochemical, and immunogenic analysis of E3 particles. **a** Surface representations of E3 F-, EC-, and EE-particles and thin slices of the corresponding central sections viewed along the two-fold axes, respectively. The surface of the virus is colored by rainbow-color based on the distance of the viral elements from the center, starting with blue (closest) through green, yellow, and orange to red (farthest from the center). **b** Structure-based evolutionary relationship among the representative viruses from HEV-Bs: E18, echovirus 18; E3, echovirus 3; CVB1, coxsackievirus B1; E6, echovirus 6; E30, echovirus 30; CVB5, coxsackievirus B5; CVB3, coxsackievirus B3; E1, echovirus 1; CVA9, coxsackievirus A9; E11, echovirus 11. **c** Comparisons of the surface of E3 F-particle with those of other representative members of HEV-B (E18 (PDB: 6HBG), CVB3 (PDB: 4GB3), and CVA9 (PDB: 1D4M)). The color scheme is the same as in **a**. The “mesa” located at the top is marked using a black dotted square for each particle. **d** Structural details of the protomer (VP1: blue; VP2: green; VP3: red) of representative particles. The loops surrounding the canyon walls (VP1 BC loop, VP1 GH loop, and VP2 EF loop) and the loops surrounding the “mesa” structures are labeled in corresponding colors. **e** The binding affinities of E3 F-particle to 5G3 IgG estimated by SPR. **f** Neutralization of E3 by 5G3 using plaque-reduction neutralization test (PRNT). The Neut_50_ value of 5G3 was 0.34 nM. **g** Surface representation of E3-5G3 complex. The viral capsid is colored the same as in **a**, and the 5G3 Fab is colored in cyan. **h** 5G3 Fab occupancy (left) and epitopes (right) located on viral pentamer. The pentamer is shown as surface (left) and cartoon (right), respectively. Residues comprising 5G3 epitope are shown as spheres. **i** The E3–5G3 binding interface. 5G3 are shown as cartoon, while E3 is shown as surface. The residues of the VP1 C-terminal (blue) insert into the 5G3 Fab hydrophobic pocket. The change of color reflects hydrophilic or hydrophobic nature of the residues, ranging from white (hydrophilic) to cyan (hydrophobic). Residues involved in the interactions between the virus (VP1: blue, VP3: red) and 5G3 are shown as sticks, and the hydrophobic interactions are shown as surface and colored in grey. Hydrogen bonds are marked as yellow dashes. **j** Clashes between 5G3 and two E3 receptors, CD55 (left)/FcRn (right). The pentamer, 5G3 light chain, 5G3 heavy chain, receptor CD55, and receptor FcRn are colored in grey, cyan, blue, magenta, and yellow, respectively.

A structure-based phylogenetic tree constructed from the biological protomers indicates four main subgroups within HEV-Bs, among which E3, together with E6 and E30 constitutes one subgroup, lying almost equidistant from the CVBs, E18, and CVA9-representative subgroups (Fig. 1b). Although the overall structures of these HEV-Bs resemble each other, major structural features distinguishing the subgroups lie in the plateaus (also named as “mesa”) surrounding the five-fold axes (Fig. 1b). Compared with the representative HEV-Bs from the other three subgroups, E3 exhibits dense “pentagon-shaped” protrusions, while CVBs and CVA9 harbor five prominent spiraling and five discontinuous protrusions, respectively (Fig. 1c). Intriguingly, E18 is remarkably smaller with a diameter of 314 Å, differing radically from that of other HEV-Bs (~ 325 Å) (Fig. 1c). These structural variations arise from the altered conformations of several loops located at the five-fold axes, such as VP1 BC, DE, and HI loops (Fig. 1d). A number of key structural elements, including the VP1 BC, DE loops and C-terminus, the VP2 EF loop and VP3 EF, GH loops, contribute to the serotype-specific antigenic sites and construct the “canyon”, where many HEV receptors often bind (Fig. 1c, d; Supplementary Fig. S4)^1,3^. Notably, VP1 C-terminus, appearing to be the most divergent in sequence among the four subgroups in HEV-Bs (Supplementary Fig. S4), is largely disordered in the native virus structures (F-, EC-, and EE-particles), indicative of a cryptic immunogenic site, akin to the VP1 GH loop in foot-and-mouth disease virus (FMDV)^9^.

To elucidate the immunogenic characteristics of E3 F-particles, sixteen monoclonal antibodies were generated by immunizing mice. Of these, the one named 5G3, exhibited tight binding to E3 with a K_D_ of 0.25 nM as determined by surface plasmon resonance (SPR) and potent neutralizing activity against E3 (a 50% neutralizing concentration (Neut_50_) value of ~ 0.3 nM) (Fig. 1e, f). To decipher the bona fide epitopes on E3 targeted by 5G3, we determined the cryo-EM structures of 5G3 in complex with E3 F- and EE-particles at 3.9 Å and 4.1 Å, respectively (Fig. 1g; Supplementary Figs. S2b, c and S3d, e and Table S2). The overall structures of two immune complexes reveal the identical epitope and binding pattern. The higher resolution structure of E3 F-particle-5G3 was used to analyze the detailed virus–antibody interactions. 5G3 binds to the viral surface along the edges of the pentameric building blocks of the virus adjacent to two-fold axes in a similar position to that observed for R10 antibody bound to hepatitis A virus (Fig. 1h)^10^. The light chain and heavy chain variable domains contribute ~ 64.2% and ~ 35.8% of the protein–protein interactions, respectively, with the light chain predominantly binding VP1 (Fig. 1i). The 5G3 paratope comprises three complementary determining regions (CDRs): L1 (residues 31–33), L3 (residues 98 and 100), and H2 (residues 51 and 54). The 5G3 epitope contains residues 283–287 and 292 in VP1 C-terminus as well as residues 140 and 143 in VP3. Disordered in the apo structure, VP1 C-terminus (residues 275–292) in the complex structure, however, inserts into a highly hydrophobic cleft constructed by LCDR1, LCDR3, and HCDR2 (Fig. 1i, upper left panel and upper right panel). These hydrophobic interactions thereby dominate the virus–antibody recognition and this tight binding are further facilitated by a number of hydrogen bonds (Fig. 1i, lower left panel and lower right panel). Notably, none of the residues comprising the epitopes are conserved (0/7) across HEV-Bs (Supplementary Figs. S4 and S6), suggesting that 5G3 is a serotype-specific neutralizing antibody. In line with many other HEV-Bs, E3 utilizes CD55 for viral attachment and FcRn for viral uncoating, gaining efficient entry into the host cells^11^. To dissect the neutralization mechanism of 5G3, we generated the footprint of this antibody on the E3 surface and discovered that it did not overlap with the viral receptor-binding sites (CD55 and FcRn) (Supplementary Fig. S7). However, superimposition of the structures of E30–FcRn (PDB:7C9V) or E30–CD55 (PDB:7C9W) over E3–5G3 revealed steric clashes between the two receptors and 5G3 (Fig. 1j). Therefore, 5G3 could potently neutralize E3 infection via blocking the interactions with its two receptors.

In summary, the atomic structures of E3 mature virion and the two types of empty particles reveal serotype-specific structural features between the subgroups within HEV-Bs and underline the potential of E3 F- or EC-particles as ideal vaccine candidates. The immunogenic characteristics and bona fide epitopes defined by the first E3-neutralizing antibody 5G3 underpin the immunogenic differences in EV-B, informing strategies for vaccine and therapeutics design against this anicteric hepatitis associated echovirus.

## Supplementary information

Supplementary Information

## Data Availability

The atomic coordinates of E3 F-, EC- and EE-particles as well as E3 (F-particle)-, E3 (EE-particle)-5G3 complexes have been submitted to the Protein Data Bank with accession numbers: 7C9X, 7EAI, 7EAH, 7EAK, and 7EAJ, respectively. Their corresponding cryo-EM maps have been deposited in the Electron Microscopy Data Bank under accession codes: EMD-30320, EMD-31044, EMD-31043, EMD-31046, and EMD-31045, respectively. All the other materials and data involved in this study will be available on request.
