# Supplementary material for: Structural basis for neutralization of an anicteric hepatitis associated echovirus by a potent neutralizing antibody
Source: Cell Discov. 2021 May 25;7:35. doi: 10.1038/s41421-021-00264-3 (PMC8149713; doi:10.1038/s41421-021-00264-3)
Supplement: Supplementary file 1 — Supplementary Information [file 41421_2021_264_MOESM1_ESM.pdf]

Supplementary Materials for

Structural basis for neutralization of an anicteric hepatitis associated echovirus by a potent neutralizing antibody

Authors: Rui Feng[1,2]*, Lei Wang[1,2]*, Dawei Shi[3]*, Binyang Zheng[4], Li Zhang[4], Hai Hou[5], Deju Xia[3] Lunbiao Cui[4], Xiangxi Wang[1,2#], Sihong Xu[3#], Kang Wang[1#], Ling Zhu[1#]

This PDF file includes:

Methods and Materials

Figures S1 to S7

Tables S1 to S2

References

Methods and Materials

E3 production and purification

E3, obtained from Jiangsu Provincial Center of Disease Control and Prevention, was produced in RD cells by inoculating the virus at a multiplicity of infection (MOI) of 0.001. Cells were cultured in Dulbecco's modified Eagle's medium (DMEM; Sigma) supplemented with 2% fetal bovine serum (FBS) (Gibco). The cell cultures were collected 48 hours after infection and subjected to lysis by three rounds of freezing and thawing. 1% NP-40 was added to facilitate the release of the virus from the cells. The lysates were centrifuged at 3,000 rpm at 4 ℃ for 20 min to remove cell debris. Viral particles were pelleted by 20% (w/v in PBS) sucrose cushion centrifugation. The sediments containing the E3 particles were resuspended in PBS and loaded onto a continuous 15 to 45 % (w/v in PBS) sucrose density gradient and centrifuged at 29,000 rpm for 5 h. Two sets of fractions were collected and dialyzed against PBS buffer. Particles from these fractions were imaged by negative staining electron microscopy and cryo-EM analysis.

Cryo-EM and data collection

To prepare cryo-grids, 3 μl aliquots of the purified E3 virus containing the F-, EE- and EC-particles (~ 2 mg mL^{-1}) were adhered onto fresh glow-discharged 400-mesh carbon-coated copper grids (C-flat, CF-2/1-2C, Protochips). Grids were blotted for 3 s in 100% relative humidity and followed by plunge-freezing (Vitrobot, FEI) in liquid ethane. For the cryo-EM sample of the E3-5G3 Fab complex, we incubated purified E3 particles and 5G3 Fab with a molar ratio of 1:180 on ice for 10 s. Aliquots of the E3-5G3 complex were loaded onto fresh grids and freezingly plunged as above described. Cryo-EM data sets were collected on an Arctica electron microscope (FEI) operated at 200 kV. Movies (25 frames, each 0.2s, total dose of 30 e^-/A^2) were acquired by using a K2 detector with a defocus range of 1.2 to 2.5 um. The final data were collected and calibrated, resulting in a pixel size of 1.347 Å for further data processing.

Image processing

During data collection, 1,071 micrographs were recorded for the mixture of the E3 F-particles, EE-particles and EC-particles and 1,742 micrographs were recorded for the E3-5G3 mixture.

Micrographs with visible CTF rings beyond 1/5 Å in their spectra were selected for further processing. The defocus value for each micrograph was determined by using Gctf, which estimate the contrast transfer function (CTF) parameters for drift corrected micrographs, and micrographs with significant astigmatism or drift were discarded. Particles were picked manually by RELION. A total of 101,311 particles were used for 2D classification and 3D classification. Three major classes (F-, EE- and EC-particles) are picked out and used for further high-resolution refinement. Finally, 41,508 E3 F-particles, 21,394 EE-particles and 2,330 EC-particles were refined with icosahedral symmetry imposed in RELION[12,13]. The final resolution was evaluated using the gold-standard Fourier shell correlation. Map sharpening was performed by using the post processing function in RELION and the local resolution was evaluated by Resmap[14]. The reconstruction strategy for E3-5G3 complex was similar to that used for *apo* E3 particles. Although the overall resolution for the complex structures (F-particle-5G3 and EE-particle-5G3) are up to 3.9 Å, the maps for the binding interface between 5G3 and the capsid are quite weak due to the conformational heterogeneity and low occupancy of the 5G3. To improve the resolution for the binding interface, we used the block-based reconstruction strategy[15-17] for focusing classification and refinement. The orientation parameters of each particle determined in Relion were used to guide extraction of the block region (~30% bigger than 2 copies of protomer-Fab) at two-fold axes and these blocks were further 3D classified. Due to the proximity of two adjacent 5G3 Fab fragments at two-fold axes, these two Fab fragments may clash sterically, theoretically allowing single 5G3 Fab bound at two-fold axes. Despite observation of weak densities for two partial Fab fragments in 3D classification with C1 symmetry imposed, that possibly was caused by the quasi-equivalent two-fold averaging under the two-fold axis microenvironment. A local reconstruction focusing on the class with single Fab bound was carried out, yielding a resolution of 3.9 Å.

Model building and refinement

First, the atomic structure of E30 full particle (PDB code 7C9S) was used as a homology model to be manually fitted into the cryo-EM map of E3 using Chimera[18], and was corrected according to the sequence of E3 capsid proteins in COOT. The resulting atomic model of E3 F-particle was

further improved in a pseudo-crystallographic manner by iterative positional and B-factor refinement in real space using Phenix and re-corrected in COOT[19,20] against the cryo-EM maps. The structure of E3 EE- and EC-particles were obtained using the same method described above. The heavy chain and the light chain of the structure of 5G3 Fab fragment were derived from H263.A2, a MAb against HPV16 (PDB code 3J8W) and H16.U4, a MAb against HPV16 (PDB code 3JBA) respectively with the same method and combined with the *apo* E3 structure to finally obtain the atomic structure of the complex.

Monoclonal antibodies preparation

The purified inactivated E3 F-particles were mixed 1:1 with Freund's complete adjuvant and injected intraperitoneally (ip) into BALB/c mice at a concentration of 50 μg/mice. These mice were administered another three booster doses with an interval of two weeks between each injection. Five days after the last boosting, the spleen cells of the mice were removed and fused with SP2/0 cells to generate hybridomas, which was followed by cultivation and selection in HAT medium and HT medium. Finally, the positive hybridomas were screened by ELISA where the purified E3 particles were used as coating antigen and each supernatant obtained from the selected hybridomas was used as the primary antibody to screen the ones capable of binding with E3 specifically. After that, the positive clones were mixed with E3 virions [plaque-forming unit (PFU) ranged from 50 to 100] at 37 ℃ for 1 h and then added onto the RD cell monolayers to screen the ones that could neutralize E3 by plaque-reduction neutralization test (PRNT) as described below.

Production of Fab fragments

MAb 5G3 was purified from mouse ascites by using protein A affinity column (GE) and the Fab fragments were purified by using Pierce FAB preparation kit (Thermo Scientific) following the manufacturer's instructions and as described previously[21]. Briefly, the sample was first desalted by desalting column and then mixed with papain to cleave the Fab fragments from the whole IgG. The Fab fragments were then separated from the Fc using Protein A.

Surface plasmon resonance

The purified E3 F-particles were immobilized onto a CM5 sensor chip surface using the NHS/EDC method to a level of ~800 response units (RUs) using Bia-core T100 (GE Healthcare) and PBS running buffer (containing 0.05% Tween-20). The gradient concentrations of purified 5G3 IgG flew over the chip at a rate of 20 μl/min. After each cycle, the chip was regenerated by 10 mM glycine-HCl (pH 1.5). The response of the antibody to the E3 virions was recorded at room temperature and the data was analyzed using Bia-core T100 Evaluation Software (GE Healthcare).

Plaque-reduction neutralization test

The 5G3 IgG was diluted in Dulbecco's modified Eagle's medium (DMEM) to obtain two-fold serial dilutions with the highest concentration at 128 nM. The same amounts of E3 virions (PFU = 90) were mixed with the prepared dilutions or PBS incubated at 37 ℃ for 1 h and dropped onto the confluent monolayers of RD cells seeded in 6-well plates. The plates were placed in the 5% CO_2 cell incubator and rocked gently every 20 min for 1 h. Afterwards, they were rinsed with DMEM (pH 7.4) for three times. The wells were then covered with the agarose overlay (2 mL/well) supplemented with 2% FBS and incubated in the cell incubator for another three days. Plaques were visualized by staining with 2.5% crystal violet and the percent inhibition was calculated as $(N_{control} - N_{test})/ N_{control} \times 100\%$, where $N_{control}$ and N_{test} represent the mean of plaque counts observed in the control group and test group, respectively. All experiments were performed in triplicate.

Analytical ultracentrifugation (AUC)

Sedimentation velocity experiments were performed on a Beckman XL-I analytical ultracentrifuge at 20 °C. Samples containing full and empty particles were diluted with PBS buffer (pH 7.4) to 400 μL with A280 nm absorption of 0.6 and further loaded into a conventional double-sector quartz cell and mounted in a Beckman four-holeAn-60Ti rotor. Data were collected at 8,064 × g at a wavelength of 280 nm and the interference sedimentation coefficients were calculated using the SEDFIT software program

Supplementary Figures

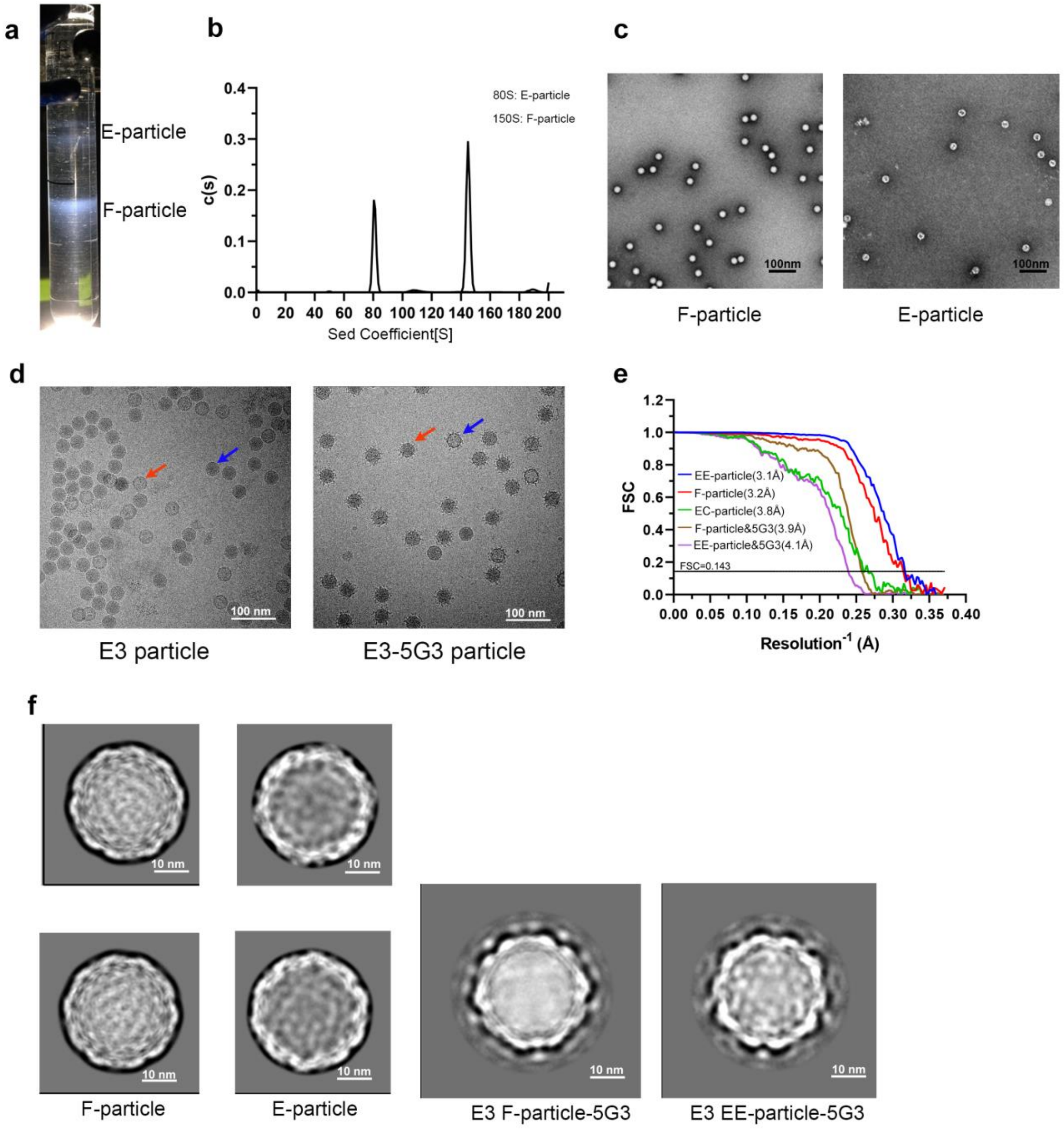

Supplementary Fig. S1. Purification and characterization of E3 and 5G3 Fab, cryo-EM images and analysis of the map resolution. (a) Sucrose density gradient (15% to 45%) ultracentrifugation for the purification of E3. Two bands were observed - the upper fraction with empty particles (E-particles), and the lower one with F-particles. (b) Analytical ultracentrifugation. The purified virus samples yielded two major peaks, 80S and 150S, which corresponded to E3 E-particles and F-particles, respectively. (c) The negative stain images of E3

F-particles (left) and E-particles (right). (d) Cryo-EM images of E3 F-particles and E-particles (left), and E3 particles in complex with 5G3 Fab (right). (e) Gold-standard Fourier shell correlation (FSC) curves of the final maps of E3 F-particle, EC-particle, EE-particle and F-particle in complex with 5G3 Fab. (f) Representative classes from 2D classifications of each structure in Relion 3.0.

a

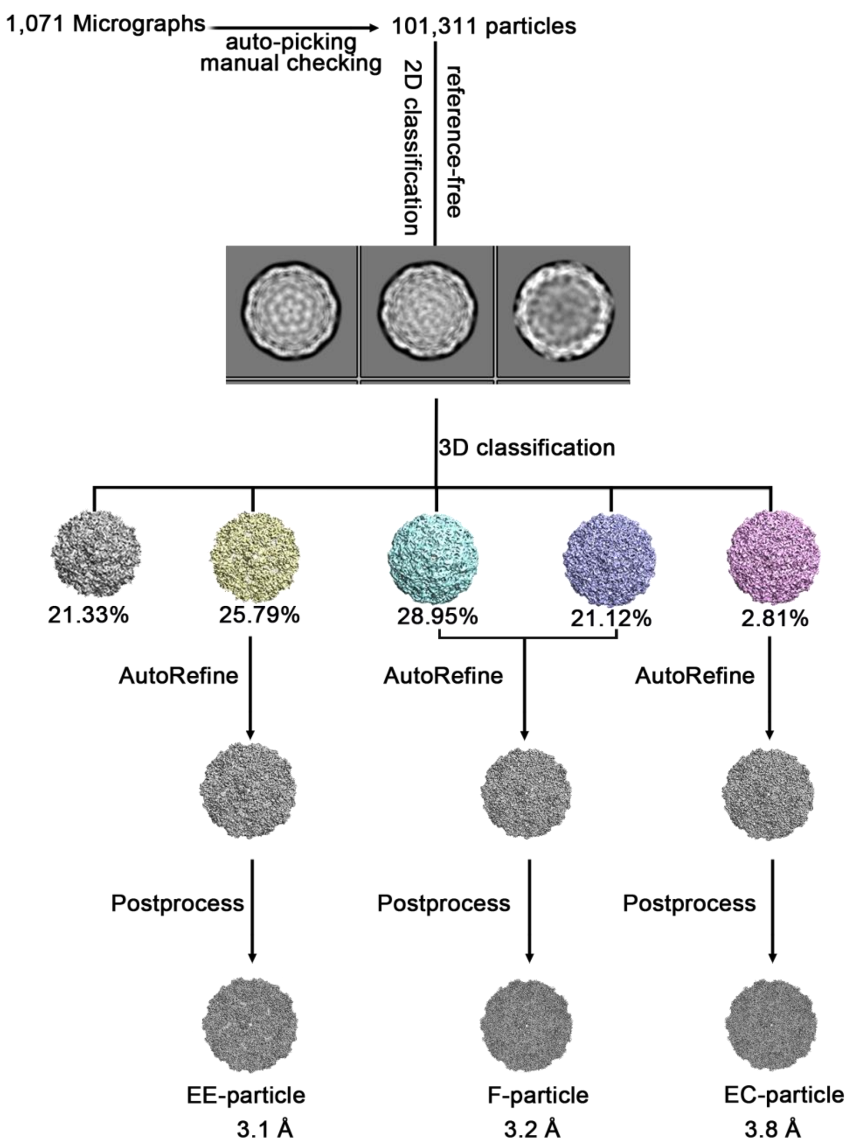
1,071 Micrographs
auto-picking
manual checking
101,311 particles
2D classification
reference-free
3D classification
21.33%
25.79%
28.95%
21.12%
2.81%
AutoRefine
AutoRefine
AutoRefine
Postprocess
Postprocess
Postprocess
EE-particle
3.1 Å
F-particle
3.2 Å
EC-particle
3.8 Å

b

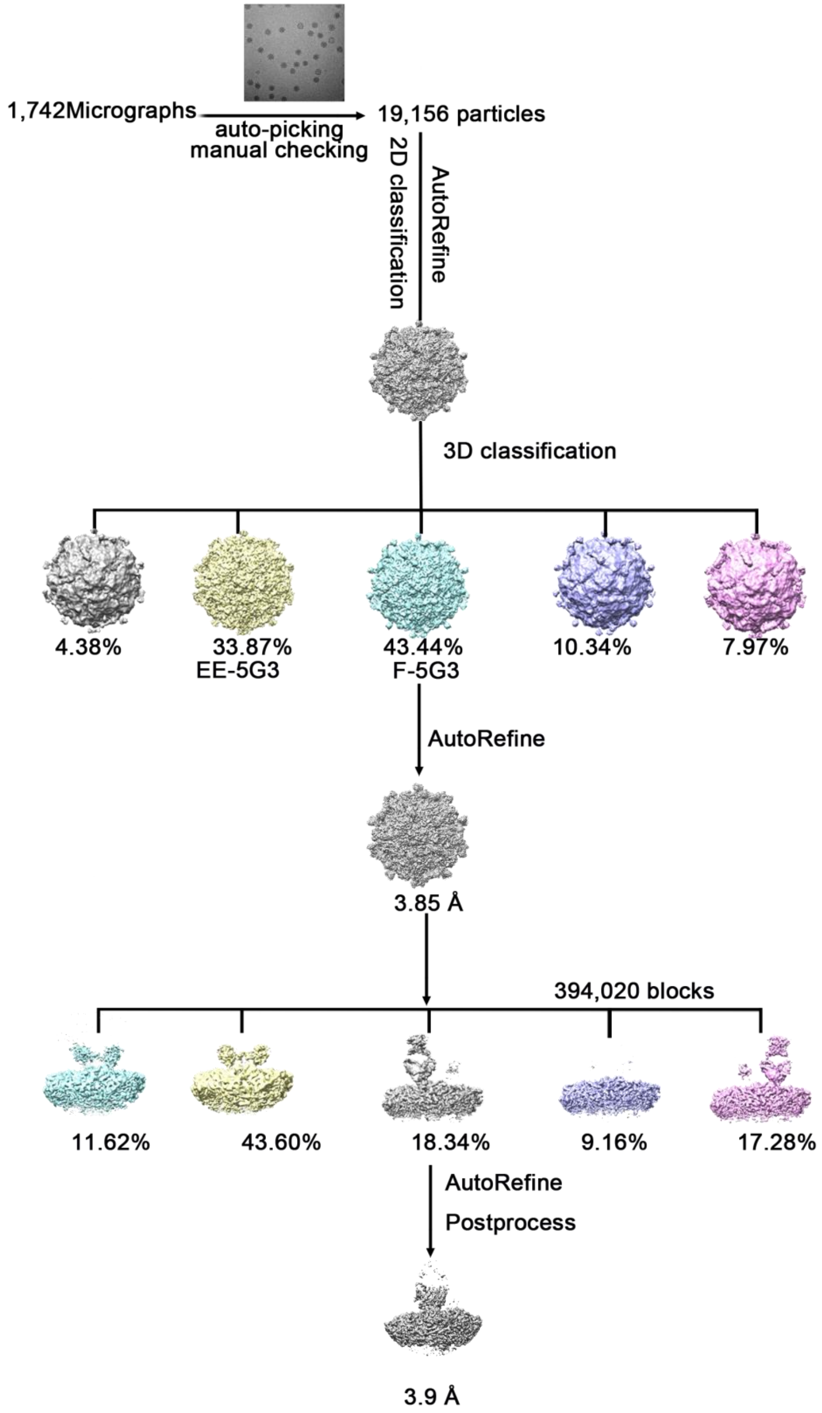
1,742Micrographs
auto-picking
manual checking
19,156 particles
2D classification
AutoRefine
3D classification
4.38%
33.87%
EE-5G3
43.44%
F-5G3
10.34%
7.97%
AutoRefine
3.85 Å
394,020 blocks
11.62%
43.60%
18.34%
9.16%
17.28%
AutoRefine
Postprocess
3.9 Å

c

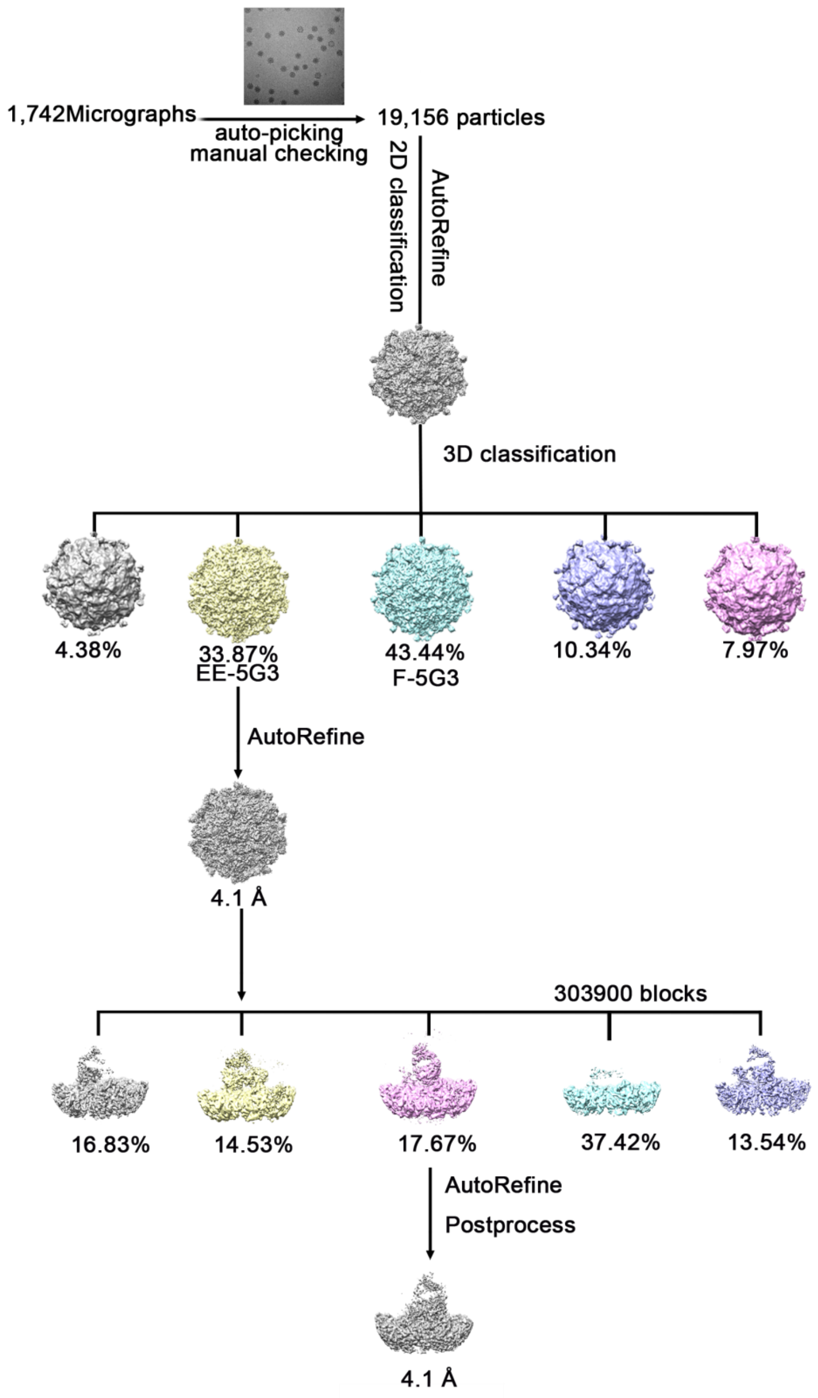

Supplementary Fig. S2. Flow-chart of cryo-EM data processing. (a) , (b) and (c) show the data processing of E3 particles, E3 F-particles in complex with 5G3 Fab and E3 EE-particles in complex with 5G3 Fab, respectively.

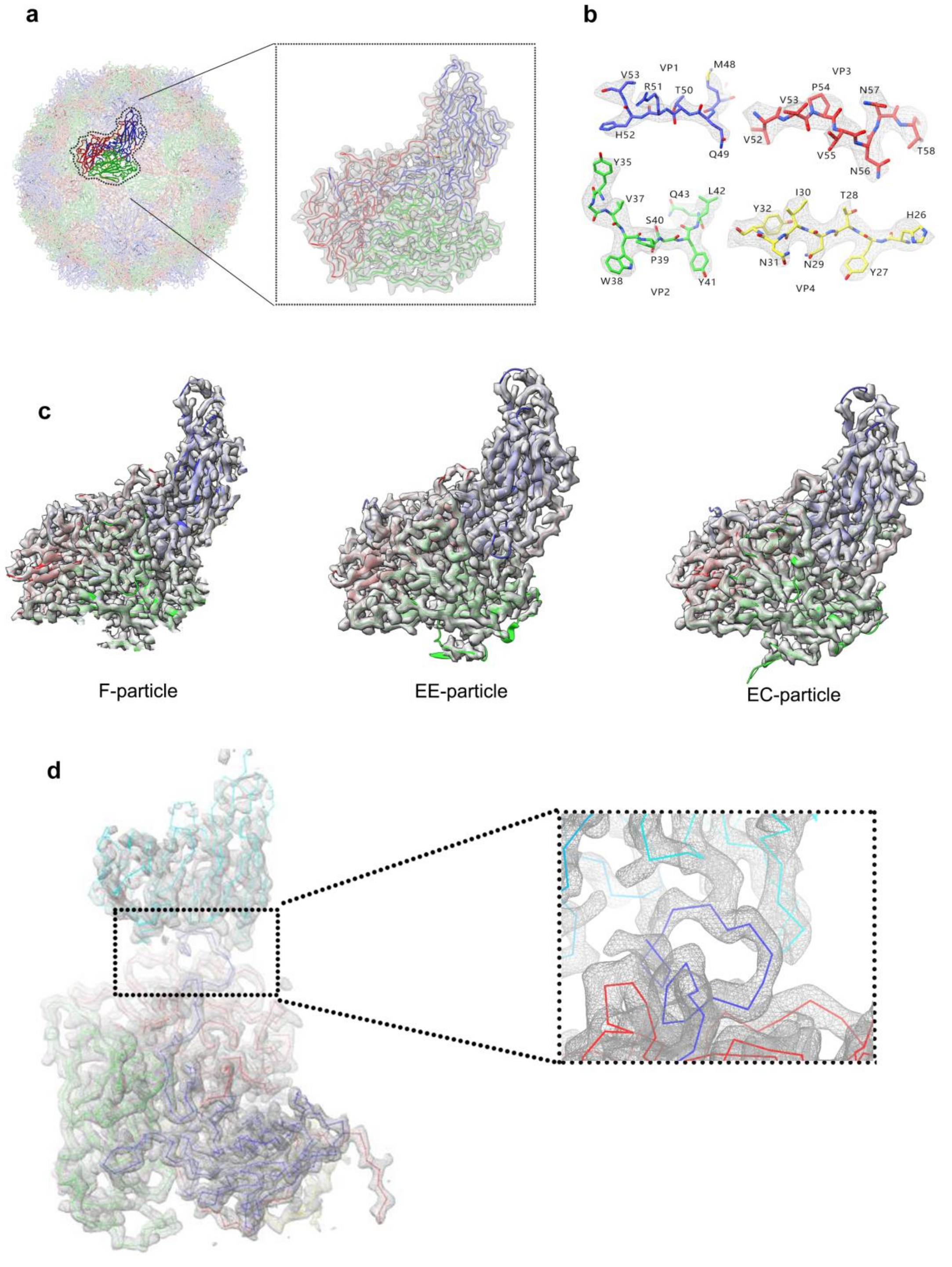
a
b
VP1
M48
V53
R51
T50
H52
Q49
VP3
P54
N57
V53
V52
V55
N56
T58
Y35
V37
Q43
L42
S40
P39
W38
Y41
VP2
Y32
I30
T28
H26
N31
N29
Y27
VP4
c
F-particle
EE-particle
EC-particle
d

182

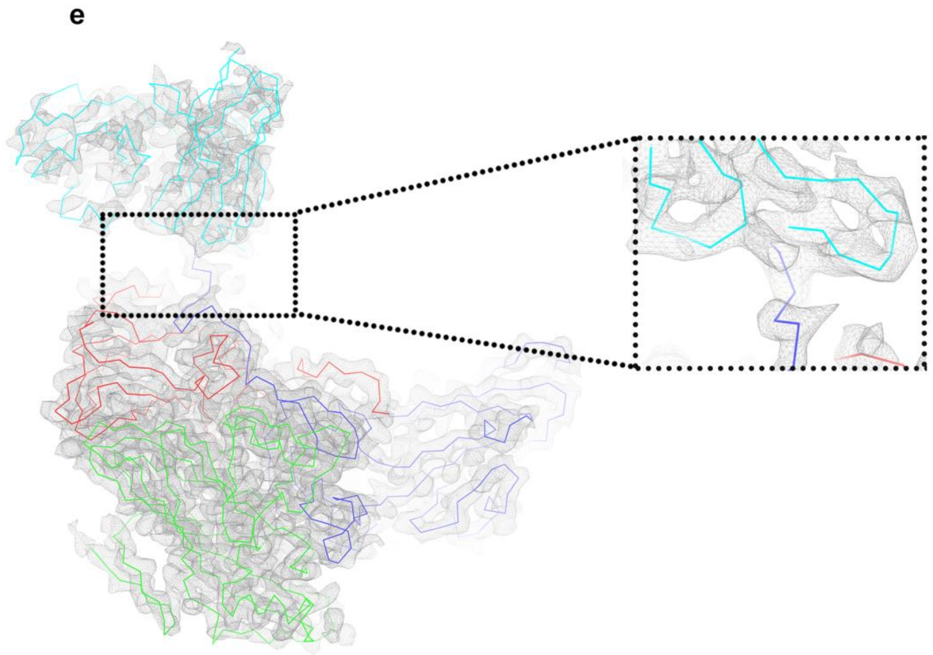

Supplementary Fig. S3. (a) Cartoon representation of the E3 F-particle perceived from the two-fold axes (left). A protomer is shown as line and accentuated (right). The signature colors (VP1: blue; VP2: green; VP3: red; VP4: yellow) are assigned as shown. (b) The cryo-EM maps of section of VP1, VP2, VP3 and VP4, respectively. (c) The cryo-EM maps of E3 F-particle, EE-particle and EC-particle, respectively. (d) The cryo-EM map of the overall E3-5G3 Fab complex (left) and the zoom-in binding interface between E3 and 5G3 Fab (right). (e) The cryo-EM map of the overall E3-EE particle -5G3 Fab complex (left) and zoom-in binding interface between E3 EE-particle and 5G3 Fab (right).

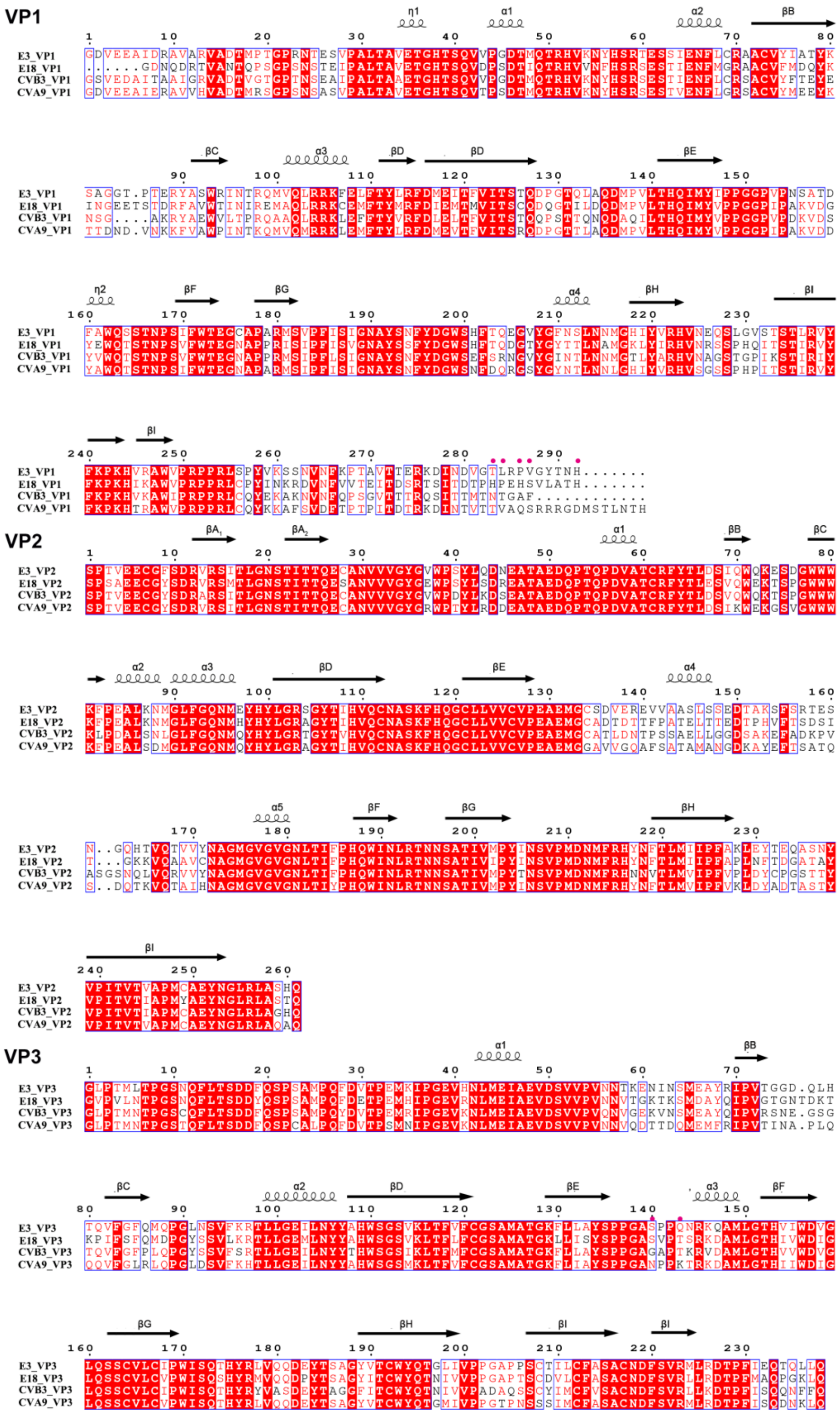
VP1
E3_VP1
E18_VP1
CVB3_VP1
CVA9_VP1
VP2
E3_VP2
E18_VP2
CVB3_VP2
CVA9_VP2
VP3
E3_VP3
E18_VP3
CVB3_VP3
CVA9_VP3

Supplementary Fig. S4. ESPript representation of sequence alignments of representative HEV-Bs. The sequences of E3 structural proteins were aligned with the other typical members (E18, CVB3 and CVA9) of enterovirus B. The residues constituting the epitopes of 5G3 are marked with dots in magenta.

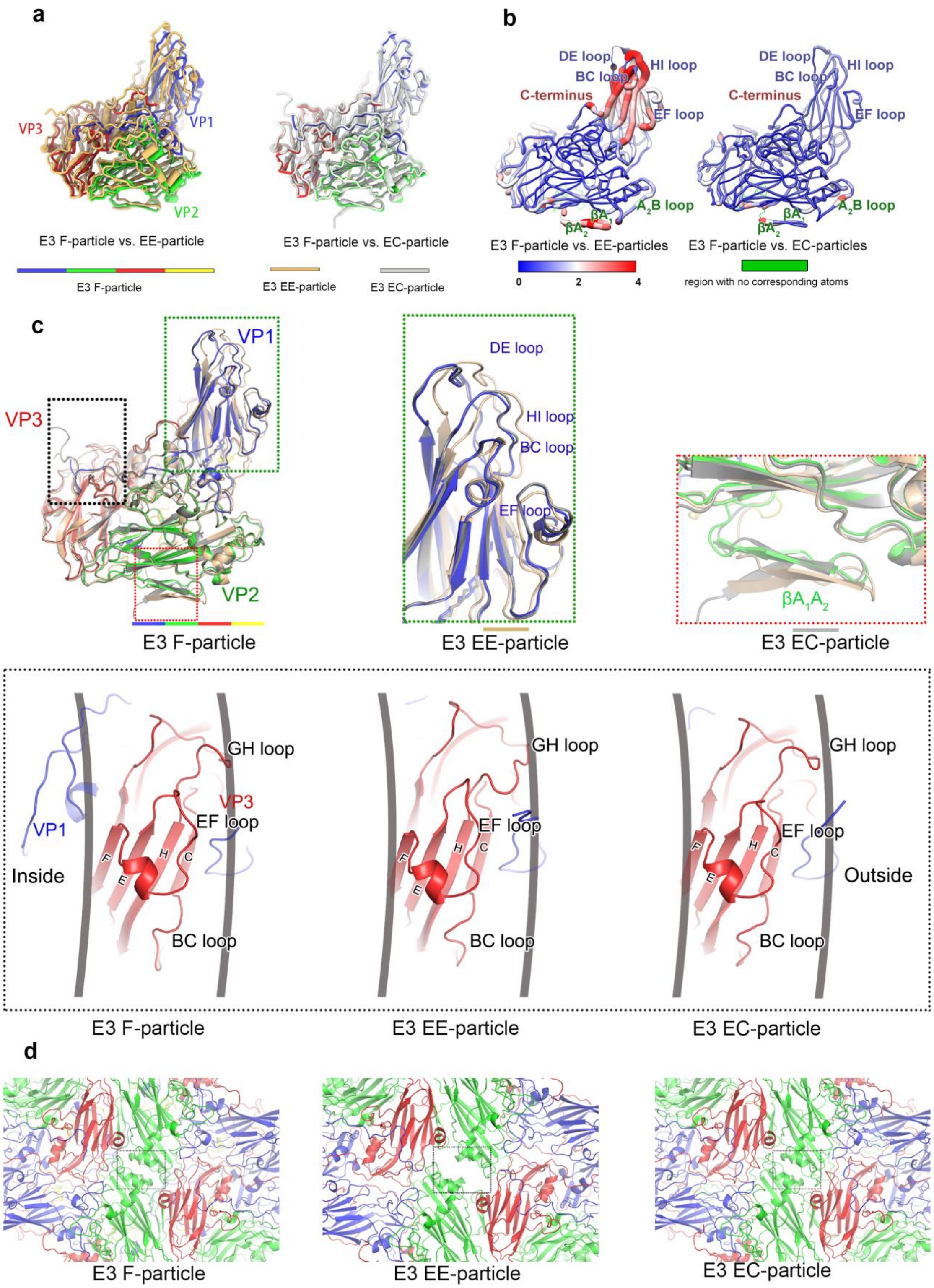

206 **Supplementary Fig. S5.** (a) Comparison of a single icosahedral protomer of the E3 F-particle

(VP1: blue; VP2: green; VP3: red; VP4: yellow) with EE- (wheat) and EC- (grey) particles. The promoters are shown in cartoon representation. (b) Superposition of the F-particle over EE-particle (left) and F-particle over EC-particle (right) reveal the difference of the structure in the protomer of the full particle; the changes of color reflect the local deviation between the structures [from blue (0 Å) to red (4 Å)]. (c) Superposition of the individual units from E3 F- (VP1: blue; VP2: green; VP3 ： red), EE- (wheat) and EC-particle (grey). The major differences among the three types of particles in VP1, VP2 are emphasized in the colored frames, the structural alterations in VP3 are emphasized in grey inset. (d) The views of the center of the two-fold axes of E3 F-(left), EE-(middle) and EC-particle (right). The region marked in black dotted rectangle, probably representing a channel, is closed in F- and EC-particles, while the counterpart in EE-particle is open.

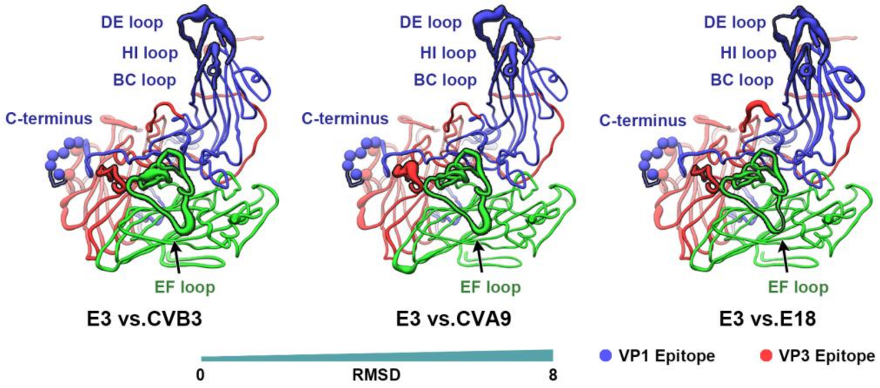

Supplementary Fig. S6. Comparison of the structural differences between E3 F-particle and those from other representative viruses of HEV-Bs. The thickness of the loop reflects the r.m.s.d between the structural elements (from thin (0) to thick (8)). The VP1 and VP3 epitopes are marked with dots in blue and red, respectively.

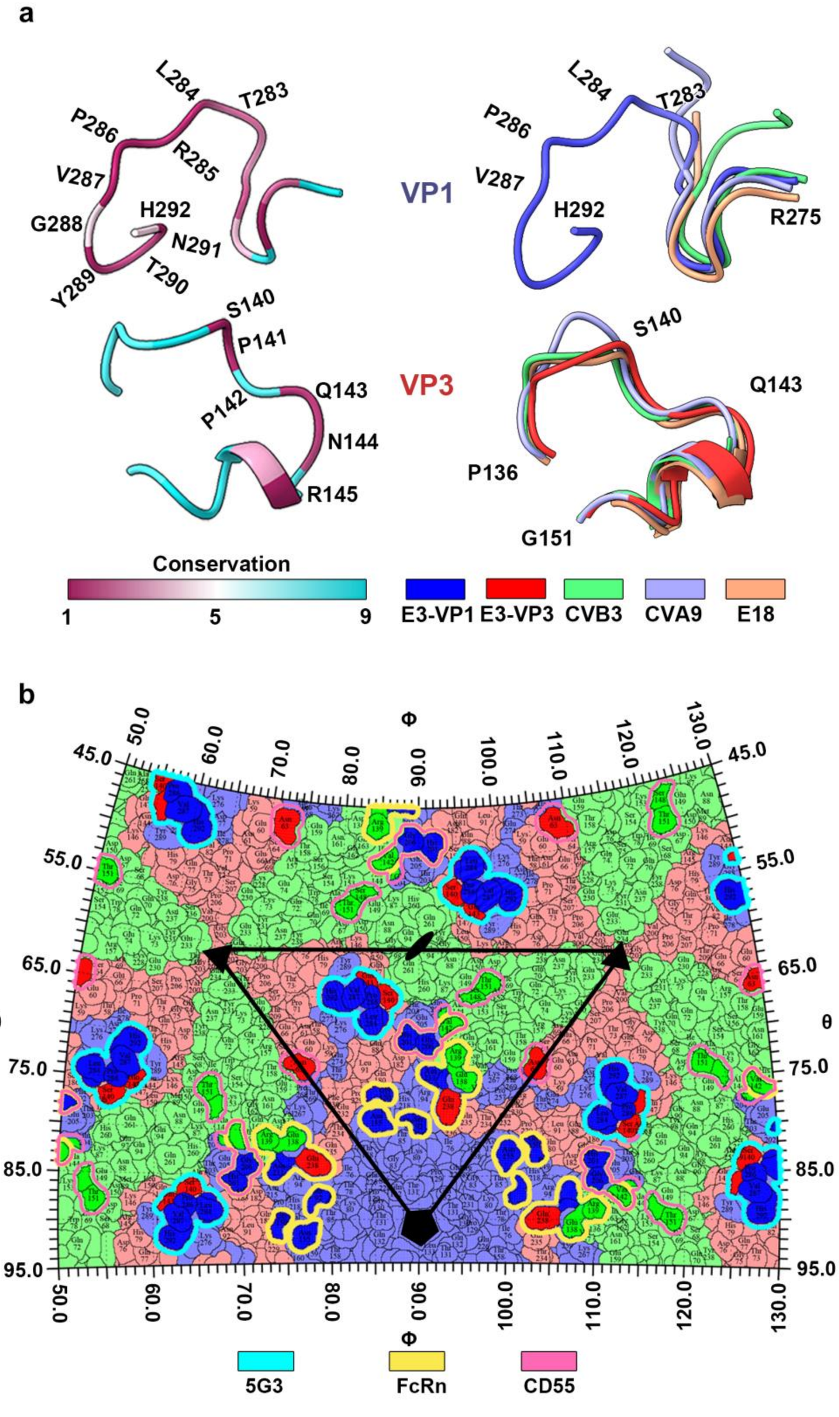
a
L284
T283
P286
R285
V287
H292
G288
N291
Y289
T290
VP1
L284
T283
P286
V287
H292
R275
S140
P141
Q143
P142
N144
R145
VP3
S140
Q143
P136
G151
Conservation
1
5
9
E3-VP1
E3-VP3
CVB3
CVA9
E18
b
Φ
θ
5G3
FcRn
CD55

Supplementary Fig. S7. (a) The left panel represents the sequence conservation analysis. The residues from E3 which are involved in the interactions between E3 and 5G3 are aligned with the corresponding residues from other representative HEV-Bs (E18, CVB3, CVA9), and colored according to the sequence conservation as listed below. The right panel depicts the structural conservation analysis. The VP1 C-terminus (blue) and VP3 EF loop (red) are superposed with their counterparts from E18 (wheat), CVB3 (light green), and CVA9 (taro purple). (b) The 5G3 footprints on the E3 surface. A two-dimensional projection of the E3 surface was produced using RIVEM. Residues of VP1, VP2, VP3 are colored in pale blue, pale green, pale red, respectively. Residues involved in the binding to 5G3 are shown in bright colors corresponding to the domain to which they belong to. The footprints of 5G3 and receptor units are outlined in cyan, yellow and hot pink respectively. Five-, three-, and two-fold icosahedral symmetry axes are marked.

Supplementary Table S1. Cryo-EM data collection and refinement statistics

Protein	F-particle	EC-particle	EE-particle	E3-5G3	EE-5G3
Magnification	22,400	22,400	22,400	22,400	22,400
Voltage (kV)	200	200	200	200	200
Electron dose ($e^-/Å^2$)	30	30	30	30	30
Defocus range (μm)	1.2-2.5	1.2-2.5	1.2-2.5	1.2-2.5	1.2-2.5
Pixel size (Å)	1.347	1.347	1.347	1.312	1.312
Symmetry imposed	I3	I3	I3	C1	C1
Detector	K2	K2	K2	K2	K2
Initial particle images (no.)	101,311	101,311	101,311	19,156	19,156
Final particle images (no.)	41,508	2,330	21,394	70,559	41,237
Map resolution (Å)	3.2	3.8	3.1	3.9	4.1
FSC threshold	0.143	0.143	0.143	0.143	0.143
Map resolution range (Å)	3-6	3-6	3-6	3-6	3-6
**Refinement**					
Initial model used (PDB code)	7C9S	7C9U	7C9U	7C81	7C81
Model resolution (Å)	2.9	3.4	3.4	3.1	3.1
FSC threshold	0.143	0.143	0.143	0.143	0.143
Model resolution range (Å)	2.9	3.4	3.4	3.1	3.1
Map sharpening B factor ($Å^2$)	-146.654	-128.713	-155.699	-127.373	-143.873
Model composition					
Non-hydrogen atom	6,455	5,986	5,596	8,291	7,403
Protein residues	820	758	710	1,056	943
B factors ($Å^2$)					
Protein	43.91	126.90	40.15	79.59	58.18
Ligand	42.66	124.78	NA	78.77	NA
R.m.s.d					
Bond lengths (Å)	0.007	0.008	0.008	0.007	0.009
Bond angles (°)	1.145	1.330	1.295	1.129	1.345
Validation					
MolProbity score	1.69	2.11	1.78	2.18	2.34
Clashscore	5.27	12.34	5.19	16.30	21.21

Poor rotamer (%)	0.14	0.00	0.16	0.22	0.24
Ramachandran statistics					
Favored (%)	93.95	91.31	91.62	92.51	91.10
Allowed (%)	5.93	8.56	8.24	7.29	8.68
Outliers (%)	0.12	0.13	0.14	0.19	0.21

Supplementary Table S2. Residues of 5G3 Fab fragment interacting with E3 (d < 4 Å)

E3 F-particle			5G3 Fab	
**Location**	**Domain**	**Residues**	**Light chain**	**Heavy chain**
**VP1**	**C-terminus**	T283	Y31	
		L284	Y98	
		P286	Y31, Y38, Y98	
		V287	Y100	
		H292		Y51, N54
**VP3**	**EF loop**	S140	S32	
		Q143	Y31, N33	

Reference

12 Scheres, S. H. Processing of Structurally Heterogeneous Cryo-EM Data in RELION. *Methods in enzymology* **579**, 125-157 (2016).

13 Zhang, K. Gctf: Real-time CTF determination and correction. *J Struct Biol* **193**, 1-12 (2016).

14 Kucukelbir, A., Sigworth, F. J. & Tagare, H. D. Quantifying the local resolution of cryo-EM density maps. *Nat Methods* **11**, 63-65 (2014).

15 Yang, Y. *et al.* Architecture of the herpesvirus genome-packaging complex and implications for DNA translocation. *Protein & cell* **11**, 339-351 (2020).

16 Wang, N. *et al.* Architecture of African swine fever virus and implications for viral assembly. *Science* **366**, 640-644 (2019).

17 Wang, N. *et al.* Structures of the portal vertex reveal essential protein-protein interactions for Herpesvirus assembly and maturation. *Protein & cell* **11**, 366-373 (2020).

18 Yang, Z. *et al.* UCSF Chimera, MODELLER, and IMP: an integrated modeling system. *J Struct Biol* **179**, 269-278 (2012).

19 Emsley, P. & Cowtan, K. Coot: model-building tools for molecular graphics. *Acta crystallographica. Section D, Biological crystallography* **60**, 2126-2132 (2004).

20 Afonine, P. V. *et al.* Towards automated crystallographic structure refinement with phenix.refine. *Acta crystallographica. Section D, Biological crystallography* **68**, 352-367 (2012).

21 Qiu, X. *et al.* Structural basis for neutralization of Japanese encephalitis virus by two potent therapeutic antibodies. *Nature Microbiology* **3**, 287-294 (2018).
